# Supplementary material for: Low doses of the organic insecticide spinosad trigger lysosomal defects, elevated ROS, lipid dysregulation, and neurodegeneration in flies
Source: eLife. 2022 Feb 22;11:e73812. doi: 10.7554/eLife.73812 (PMC8863376; doi:10.7554/eLife.73812)
Supplement: Figure 6—source data 1. — Lipidomic profile of larvae exposed to 2.5 parts per million (ppm) spinosad or control (equivalent dose of dimethyl sulfoxide) for 2 hr as detected by liquid chromatography-mass spectrometry. Values are expressed as peak intensity area normalized to sample weight. [file elife-73812-fig6-data1.docx]

**Figure 6 – Source Data 1. Impact of spinosad on the lipidomic profile.** Lipidomic profile of larvae exposed to 2.5 ppm spinosad or control (equivalent dose of DMSO) for 2 hr as detected by LC-MS. Values are expressed as peak intensity area normalized to sample weight.

| Lipid species | Control 1 | Control 2 | Control 3 | Spinosad 1 | Spinosad 2 | Spinosad 3 | ANOVA,  Tukey’s HSD  p-adj | F-value |
| --- | --- | --- | --- | --- | --- | --- | --- | --- |
| 2HPOT keto 34:2-PE-/16:0 | 123795.62 | 163589.74 | 219673.91 | 217767.86 | 176250 | 175247.52 | 0.5419233 | 0.443 |
| 2HPOT keto 34:2-PG-/16:0 | 90656.9 | 67008.5 | 89021.7 | 104107.1 | 117589.3 | 76435.6 | 0.2970207 | 1.435 |
| 2HPOT keto 34:3-PC-/16:0 | 77372.3 | 33076.9 | 58043.5 | 41875 | 42589.3 | 55247.5 | 0.5176349 | 0.502 |
| 2HPOT keto 34:3-PE-/16:0 | 933065.69 | 952820.51 | 1215326.1 | 1248660.7 | 1132232.1 | 1204851.5 | 0.1715842 | 2.767 |
| 2HPOT keto 34:3-PG-/16:0 | 778321.2 | 873846.2 | 713152.2 | 958750 | 810982.1 | 959703 | 0.1486808 | 3.189 |
| 2HPOT keto 36:4-PC-/2HPOT keto 36:4 | 754744.5 | 938119.7 | 916087 | 849821.4 | 981875 | 931584.2 | 0.4999316 | 0.549 |
| 2HPOT keto 36:4-PE-/18:1 | 2131167.9 | 2425726.5 | 2663478.3 | 2802767.9 | 2864107.1 | 3127920.8 | 0.0458895 | 8.185 |
| 2HPOT keto 36:4-PE-/18:2 | 1160292 | 1192649.6 | 1200434.8 | 1141696.4 | 1364821.4 | 1291485.1 | 0.2892342 | 1.490 |
| 2HPOT keto 36:4-PG-/18:1 | 1165255.5 | 1236239.3 | 1200108.7 | 1235000 | 1367410.7 | 1312079.2 | 0.07464646 | 5.743 |
| 2HPOT keto 36:4-PG-/18:2 | 667737.2 | 667435.9 | 531521.7 | 607232.1 | 672142.9 | 596534.7 | 0.9549847 | 0.004 |
| 2HPOT keto 36:5-PC-/18:3 | 680438 | 810940.2 | 648260.9 | 814821.4 | 845357.1 | 599108.9 | 0.6872676 | 0.188 |
| 2HPOT keto 36:5-PE-/18:2 | 488905.11 | 508205.13 | 590434.78 | 505625 | 610803.57 | 570297.03 | 0.4911734 | 0.573 |
| 2HPOT keto 36:5-PG-/18:2 | 271678.8 | 318461.5 | 243152.2 | 339107.1 | 371071.4 | 333267.3 | 0.04814691 | 7.916 |
| 2HPOT keto 36:6-PC-/18:3 | 51824.8 | 27094 | 31521.7 | 25267.9 | 65178.6 | 50297 | 0.5080307 | 0.527 |
| CE 14:0 | 146788.3 | 242136.8 | 283695.7 | 45982.1 | 69464.3 | 39207.9 | 0.01419808 | 17.270 |
| CE 16:0 | 188102.2 | 186153.8 | 236195.7 | 359285.7 | 233035.7 | 312475.2 | 0.07171429 | 5.922 |
| CE 16:1 | 725912.4 | 732393.2 | 986195.7 | 524732.1 | 756964.3 | 828910.9 | 0.4255195 | 0.786 |
| CE 18:1 | 3085839.4 | 3167435.9 | 3113043.5 | 3207500 | 1493482.1 | 3164158.4 | 0.4256607 | 0.785 |
| CE 18:2 | 9927 | 79059.8 | 35978.3 | 20000 | 73928.6 | 35148.5 | 0.9601355 | 0.003 |
| CL 62:3 | 37606.838 | 41282.051 | 23152.174 | 19107.143 | 7232.1429 | 3564.3564 | 0.02952137 | 10.990 |
| CL 64:3 | 152820.51 | 159059.83 | 116304.35 | 65625 | 39553.571 | 22376.238 | 0.00544154 | 29.900 |
| CL 64:4 | 1155726.5 | 1217948.7 | 898260.87 | 609642.86 | 283660.71 | 155940.59 | 0.01131725 | 19.730 |
| CL 64:6 | 20341.88 | 18717.949 | 34239.13 | 23303.571 | 23392.857 | 16732.673 | 0.5750357 | 0.372 |
| CL 65:0 | 10341.88 | 13589.744 | 11847.826 | 9375 | 11071.429 | 13465.347 | 0.7017686 | 0.169 |
| CL 66:0 | 12478.632 | 12905.983 | 10000 | 10446.429 | 12946.429 | 10396.04 | 0.6892719 | 0.185 |
| CL 66:3 | 101538.46 | 129487.18 | 48152.174 | 8482.1429 | 8125 | 14059.406 | 0.02580067 | 11.980 |
| CL 66:4 | 575811.97 | 634786.32 | 513260.87 | 329017.86 | 146250 | 69702.97 | 0.009676818 | 21.600 |
| CL 66:6 | 44017.094 | 35470.085 | 30760.87 | 28303.571 | 13125 | 6831.6832 | 0.05036087 | 7.670 |
| CL 67:0 | 123247.86 | 114358.97 | 86413.043 | 55982.143 | 22767.857 | 19504.951 | 0.009472268 | 21.870 |
| CL 68:10 | 36495.727 | 32649.573 | 34347.826 | 31875 | 32589.286 | 25247.525 | 0.1506398 | 3.149 |
| CL 68:11 | 19743.59 | 23931.624 | 12934.783 | 3214.2857 | 4375 | 594.05941 | 0.008930132 | 22.620 |
| CL 68:3 | 24871.795 | 21111.111 | 23913.043 | 15535.714 | 25000 | 13267.327 | 0.2274407 | 2.029 |
| CL 68:4 | 90854.701 | 82564.103 | 43695.652 | 13214.286 | 16339.286 | 13861.386 | 0.01647519 | 15.800 |
| CL 68:6 | 1092222.2 | 1137692.3 | 817934.78 | 269553.57 | 172410.71 | 111881.19 | 0.001638514 | 57.190 |
| CL 69:0 | 636495.73 | 660000 | 430108.7 | 88303.571 | 80892.857 | 60891.089 | 0.002459115 | 46.080 |
| CL 70:0 | 62136.752 | 79914.53 | 57173.913 | 58392.857 | 73571.429 | 54059.406 | 0.6536651 | 0.234 |
| CL 70:10 | 111880.34 | 130085.47 | 100326.09 | 111785.71 | 111428.57 | 89108.911 | 0.4324939 | 0.760 |
| CL 70:2 | 97179.487 | 114871.79 | 105652.17 | 106160.71 | 117321.43 | 89009.901 | 0.8664366 | 0.032 |
| CL 70:4 | 5811.9658 | 14102.564 | 13369.565 | 8839.2857 | 12946.429 | 7821.7822 | 0.7108664 | 0.159 |
| CL 70:6 | 113675.21 | 110512.82 | 45760.87 | 8571.4286 | 14464.286 | 19702.97 | 0.02761109 | 11.470 |
| CL 72:10 | 30427.35 | 39658.12 | 21521.739 | 17321.429 | 15625 | 30693.069 | 0.2582735 | 1.734 |
| CL 72:11 | 52307.692 | 75555.556 | 58260.87 | 46250 | 52232.143 | 50891.089 | 0.1642574 | 2.892 |
| CL 72:4 | 214871.79 | 201452.99 | 188913.04 | 183125 | 198214.29 | 152574.26 | 0.1969343 | 2.391 |
| CL 74:7 | 100341.88 | 94957.265 | 50217.391 | 68839.286 | 63125 | 35544.554 | 0.2413577 | 1.888 |
| DG 28:0 -(14:0) | 41232263 | 43517778 | 56964783 | 48507232 | 51229643 | 39195248 | 0.8867293 | 0.023 |
| DG 30:0 -(14:0) | 20333869 | 16738120 | 33330544 | 35592321 | 31987768 | 26947426 | 0.2262944 | 2.041 |
| DG 30:0 -(15:0) | 222116.8 | 189145.3 | 224782.6 | 84910.7 | 263125 | 203861.4 | 0.6286463 | 0.273 |
| DG 30:0 -(16:0) | 31792482 | 28049829 | 52891848 | 46444821 | 47469464 | 38735743 | 0.4640578 | 0.654 |
| DG 30:1 -(14:0) | 112222774 | 95524615 | 144444891 | 135975089 | 129559554 | 93132277 | 0.9176174 | 0.012 |
| DG 30:1 -(14:1) | 13757883 | 11986325 | 20162065 | 16855982 | 15285268 | 12458911 | 0.8838297 | 0.024 |
| DG 30:1 -(16:0) | 12133212 | 11247180 | 18532283 | 15314375 | 13634464 | 10981980 | 0.8132627 | 0.064 |
| DG 30:1 -(16:1) | 141050292 | 119525299 | 179823044 | 164481964 | 152594286 | 117753762 | 0.9383107 | 0.007 |
| DG 32:0 -(14:0) | 1981678.8 | 2075042.7 | 2994347.8 | 2233750 | 2945982.1 | 2221881.2 | 0.7858322 | 0.084 |
| DG 32:0 -(16:0) | 28784891 | 24649402 | 42998804 | 38700089 | 35333304 | 30870198 | 0.6625472 | 0.221 |
| DG 32:0 -(18:0) | 4620875.9 | 3709059.8 | 6845978.3 | 6595178.6 | 5694732.1 | 5813465.3 | 0.3728798 | 1.005 |
| DG 32:1 -(14:0) | 119655329 | 117577436 | 153631848 | 146367500 | 136873125 | 118173960 | 0.8181483 | 0.060 |
| DG 32:1 -(14:1) | 1084744.5 | 1191111.1 | 2034456.5 | 1757053.6 | 1613750 | 1260891.1 | 0.7648967 | 0.102 |
| DG 32:1 -(16:0) | 93221387 | 88162821 | 125805978 | 119928393 | 110233036 | 87595347 | 0.8280745 | 0.054 |
| DG 32:1 -(16:1) | 112397080 | 104677350 | 148316522 | 139003393 | 124129732 | 102728515 | 0.9931095 | 0.000 |
| DG 32:1 -(18:0) | 892335.8 | 1543418.8 | 2525978.3 | 2191607.1 | 1834732.1 | 1679505 | 0.6448556 | 0.248 |
| DG 32:1 -(18:1) | 137173577 | 129496667 | 183220326 | 171838125 | 156348125 | 127531980 | 0.9314351 | 0.008 |
| DG 32:2 -(14:0) | 8099197.1 | 7010427.4 | 9596304.3 | 8794107.1 | 9024553.6 | 6243069.3 | 0.8626771 | 0.034 |
| DG 32:2 -(14:1) | 16463504 | 14628974 | 23230109 | 20327679 | 18999107 | 14671881 | 0.9740802 | 0.001 |
| DG 32:2 -(16:1) | 204709270 | 186125812 | 293435000 | 296117857 | 290351339 | 218629703 | 0.3861522 | 0.945 |
| DG 32:2 -(18:1) | 13110438 | 11873162 | 17568261 | 17448482 | 14924643 | 13072574 | 0.6761583 | 0.202 |
| DG 32:2 -(18:2) | 12836788 | 11616068 | 18780326 | 13928304 | 15833839 | 9151980.2 | 0.6536608 | 0.234 |
| DG 34:0 -(14:0) | 180729.9 | 139743.6 | 273369.6 | 163928.6 | 226339.3 | 141089.1 | 0.6807093 | 0.196 |
| DG 34:0 -(16:0) | 5460729.9 | 4198547 | 7729239.1 | 7772678.6 | 7168303.6 | 5416435.6 | 0.4733192 | 0.625 |
| DG 34:0 -(18:0) | 6165255.5 | 4886837.6 | 9138369.6 | 8686607.1 | 8398571.4 | 7318019.8 | 0.34947 | 1.121 |
| DG 34:0 -(20:0) | 236496.4 | 342649.6 | 233587 | 268928.6 | 169732.1 | 255544.6 | 0.4520102 | 0.693 |
| DG 34:1 -(16:1) | 9410802.9 | 5651282.1 | 12105761 | 13179107 | 11926964 | 8892475.2 | 0.3713354 | 1.012 |
| DG 34:1 -(18:0) | 7627445.3 | 4759401.7 | 7893369.6 | 7157678.6 | 7296517.9 | 8465445.5 | 0.4631568 | 0.657 |
| DG 34:1 -(18:1) | 167782993 | 140669658 | 246624239 | 242133214 | 237998482 | 200824455 | 0.2893835 | 1.489 |
| DG 34:1 -(20:0) | 131751.8 | 324615.4 | 272826.1 | 253303.6 | 267142.9 | 357326.7 | 0.4960737 | 0.559 |
| DG 34:2 -(16:0) | 8007518.2 | 6161880.3 | 9911739.1 | 7007232.1 | 8140446.4 | 6580891.1 | 0.542135 | 0.443 |
| DG 34:2 -(16:1) | 84736861 | 76823846 | 118052283 | 114529911 | 111164911 | 92316535 | 0.4242981 | 0.790 |
| DG 34:2 -(18:1) | 139355183 | 128887265 | 202773261 | 202966696 | 184341429 | 158678317 | 0.397652 | 0.895 |
| DG 34:2 -(18:2) | 10302044 | 8617435.9 | 13534022 | 11898839 | 12304911 | 8522970.3 | 0.9636001 | 0.002 |
| DG 36:0 -(16:0) | 188102.2 | 186153.8 | 236195.7 | 359285.7 | 235535.7 | 286831.7 | 0.08372604 | 5.251 |
| DG 36:0 -(18:0) | 1768759.1 | 1696837.6 | 2512173.9 | 1393660.7 | 1547053.6 | 1807920.8 | 0.2270012 | 2.034 |
| DG 36:0 -(20:0) | 269416.1 | 275213.7 | 423804.3 | 351160.7 | 163928.6 | 172970.3 | 0.3032436 | 1.393 |
| DG 36:1 -(16:1) | 725912.4 | 732393.2 | 986195.7 | 524732.1 | 756964.3 | 828910.9 | 0.4255195 | 0.786 |
| DG 36:1 -(18:0) | 8194452.6 | 3938803.4 | 14448696 | 12242500 | 9150446.4 | 9168415.8 | 0.7015448 | 0.170 |
| DG 36:1 -(18:1) | 10549781 | 11541026 | 15197283 | 15342411 | 13948036 | 11321980 | 0.5795313 | 0.363 |
| DG 36:1 -(20:0) | 800875.9 | 728547 | 360000 | 1111517.9 | 932500 | 912970.3 | 0.07718021 | 5.597 |
| DG 36:2 -(18:0) | 999051.1 | 484017.1 | 1448913 | 1336250 | 1281250 | 828019.8 | 0.623064 | 0.283 |
| DG 36:2 -(18:1) | 37264964 | 35416752 | 49351413 | 44835804 | 39012143 | 38627030 | 0.9770349 | 0.001 |
| DG 36:2 -(18:2) | 800583.9 | 786752.1 | 1056195.7 | 1178928.6 | 848928.6 | 973168.3 | 0.4116126 | 0.839 |
| DG 36:3 -(16:0) | 12043.8 | 25555.6 | 0 | 9017.9 | 11607.1 | 0 | 0.5270203 | 0.479 |
| DG 36:3 -(18:0) | 146131.4 | 88547 | 71630.4 | 89107.1 | 100178.6 | 81980.2 | 0.6405598 | 0.254 |
| DG 36:3 -(18:1) | 4758467.2 | 4183418.8 | 4991304.3 | 3499107.1 | 3485089.3 | 3721584.2 | 0.01296218 | 18.220 |
| DG 36:3 -(18:2) | 5452335.8 | 4341111.1 | 6264891.3 | 4566785.7 | 4248303.6 | 3911584.2 | 0.1323347 | 3.558 |
| DG 36:3 -(18:3) | 128686.1 | 168803.4 | 82173.9 | 41875 | 121785.7 | 81584.2 | 0.2584312 | 1.733 |
| DG 36:4 -(18:1) | 304452.6 | 177265 | 181304.3 | 261339.3 | 462232.1 | 373861.4 | 0.1130596 | 4.094 |
| DG 36:4 -(18:2) | 1052992.7 | 797008.5 | 930652.2 | 860535.7 | 743750 | 621485.1 | 0.1413992 | 3.345 |
| DG 36:4 -(18:3) | 409051.1 | 301965.8 | 489565.2 | 294464.3 | 512053.6 | 334455.4 | 0.8289335 | 0.053 |
| DG 38:1 -(18:1) | 3085839.4 | 3167435.9 | 3113043.5 | 3207500 | 1493482.1 | 3164158.4 | 0.4256607 | 0.785 |
| DG 38:1 -(20:0) | 680948.9 | 401367.5 | 922391.3 | 841339.3 | 1216517.9 | 864950.5 | 0.1886158 | 2.506 |
| DG 38:4 -(20:3) | 27226.3 | 15042.7 | 17391.3 | 9017.9 | 0 | 0 | 0.02438948 | 12.410 |
| DG 38:5 -(16:0) | 14306.6 | 28290.6 | 27826.1 | 27410.7 | 15357.1 | 0 | 0.3712702 | 1.012 |
| DG 38:5 -(20:3) | 166642.3 | 108803.4 | 97826.1 | 78750 | 146785.7 | 159505 | 0.9109031 | 0.014 |
| DG 38:5 -(22:5) | 47080.3 | 27179.5 | 35760.9 | 15089.3 | 4017.9 | 19703 | 0.03276297 | 10.270 |
| DG 38:6 -(16:0) | 464525.5 | 914359 | 253152.2 | 330446.4 | 512053.6 | 176633.7 | 0.4012172 | 0.880 |
| DG 38:6 -(22:5) | 7299.3 | 20341.9 | 49021.7 | 21071.4 | 0 | 5940.6 | 0.2974293 | 1.433 |
| dhCer 16:0 | 259416.1 | 110427.4 | 237826.1 | 239285.7 | 158035.7 | 0 | 0.4520158 | 0.693 |
| dhCer 18:0 | 27810.2 | 26410.3 | 13152.2 | 34642.9 | 60982.1 | 33366.3 | 0.1127667 | 4.103 |
| dhCer 20:0 | 18832.1 | 47350.4 | 2934.8 | 0 | 84107.1 | 13762.4 | 0.7584759 | 0.108 |
| HOD 34:2-PC-/16:0 | 874160.6 | 350854.7 | 220869.6 | 107232.1 | 606517.9 | 393465.3 | 0.6708059 | 0.210 |
| HOD 34:3-PC-/16:0 | 20799051 | 20560769 | 20739457 | 19178214 | 18878214 | 18728218 | 0.0002974 | 138.700 |
| HOD 34:3-PE-/16:0 | 50000 | 66581.2 | 12608.7 | 15625 | 41517.9 | 39802 | 0.5829498 | 0.356 |
| HOD 34:3-PG-/HOT 34:2 PG | 163503.6 | 172051.3 | 45434.8 | 97767.9 | 84464.3 | 77920.8 | 0.3843419 | 0.953 |
| HOD 36:4-PC-/18:1 | 1337518.2 | 1207777.8 | 1465108.7 | 994732.1 | 1312946.4 | 821188.1 | 0.1439454 | 3.289 |
| HOD 36:4-PC-/18:2 | 1069854 | 672393.2 | 978478.3 | 322232.1 | 762321.4 | 464356.4 | 0.0917021 | 4.881 |
| HOD 36:4-PE-/18:1 | 55401.5 | 81794.9 | 18260.9 | 41785.7 | 18750 | 52970.3 | 0.5419486 | 0.443 |
| HOD 36:4-PG-/18:1 | 643211.7 | 651453 | 213913 | 319642.9 | 365714.3 | 391584.2 | 0.3802321 | 0.971 |
| HOD 36:5-PC-/18:2 | 5985.4 | 4529.9 | 652.2 | 1964.3 | 3660.7 | 6633.7 | 0.8706898 | 0.030 |
| HOD 36:5-PC-/18:3 | 374890.5 | 550256.4 | 403260.9 | 479642.9 | 464196.4 | 567920.8 | 0.3884604 | 0.934 |
| HOD 36:5-PG-/HOT 36:4 PG | 89854 | 52564.1 | 37391.3 | 35267.9 | 38125 | 39405.9 | 0.2262988 | 2.041 |
| HOD 36:6-PC-/18:3 | 301824.8 | 122478.6 | 134239.1 | 61696.4 | 69821.4 | 32475.2 | 0.08978089 | 4.965 |
| HOT 34:2-PC-/16:0 | 16493723 | 19311880 | 20449348 | 16124554 | 14757679 | 14744753 | 0.04842608 | 7.884 |
| HOT 34:3-PC-/16:0 | 16770073 | 16078120 | 16163696 | 13224464 | 14821875 | 13685545 | 0.009686196 | 21.590 |
| HOT 34:3-PG-/16:0 | 66058.394 | 80000 | 24239.13 | 54642.857 | 65178.571 | 69009.901 | 0.7389952 | 0.128 |
| HOT 36:4-PC-/18:1 | 24306.569 | 25555.556 | 11847.826 | 53035.714 | 14107.143 | 16633.663 | 0.6100945 | 0.305 |
| HOT 36:4-PC-/18:2 | 374890.5 | 550256.4 | 403260.9 | 479642.9 | 464196.4 | 567920.8 | 0.3884604 | 0.934 |
| HOT 36:4-PG-/18:1 | 175328.47 | 196410.26 | 52608.696 | 51696.429 | 95089.286 | 92079.208 | 0.258402 | 1.733 |
| HOT 36:5-PG-/oPDA 36:4 PG | 21532.8 | 28119.7 | 5978.3 | 3125 | 5803.6 | 11287.1 | 0.1665093 | 2.852 |
| HOT 36:6-PC-/18:3 | 48686.1 | 50000 | 47065.2 | 41160.7 | 29642.9 | 46138.6 | 0.1248379 | 3.751 |
| HPOD keto 34:2-PC-/16:0 | 34817.5 | 111025.6 | 36847.8 | 52321.4 | 53035.7 | 59604 | 0.8259478 | 0.055 |
| HPOD keto 34:2-PC-/16:0 | 172627.7 | 164188 | 222826.1 | 214196.4 | 224642.9 | 185049.5 | 0.381575 | 0.965 |
| HPOD keto 34:2-PE-/16:0 | 75109.5 | 98717.9 | 65326.1 | 108839.3 | 98214.3 | 89901 | 0.1641181 | 2.894 |
| HPOD keto 34:2-PG-/16:0 | 39416.1 | 43076.9 | 21847.8 | 54910.7 | 32053.6 | 36732.7 | 0.5370217 | 0.455 |
| HPOD keto 34:3-PC-/16:0 | 6820073 | 6517350.4 | 5980000 | 5792142.9 | 5799910.7 | 6127425.7 | 0.1191336 | 3.911 |
| HPOD keto 34:3-PC-/16:0 | 660802.9 | 655982.9 | 600760.9 | 519375 | 651517.9 | 619901 | 0.3937338 | 0.912 |
| HPOD keto 34:3-PC-/18:3 | 2117226.3 | 2741880.3 | 4474565.2 | 4585982.1 | 893750 | 4329405.9 | 0.9143147 | 0.013 |
| HPOD keto 34:3-PE-/HPOT keto 34:2-PE | 627591.2 | 662735 | 574130.4 | 684642.9 | 671607.1 | 866336.6 | 0.1536639 | 3.089 |
| HPOD keto 34:3-PG-/16:0 | 537591.24 | 621196.58 | 576847.83 | 649107.14 | 732589.29 | 841386.14 | 0.05535879 | 7.170 |
| HPOD keto 36:4-PC-/18:1 | 2698686.1 | 1997948.7 | 2249891.3 | 2194375 | 2144642.9 | 2142079.2 | 0.4925564 | 0.569 |
| HPOD keto 36:4-PC-/18:1 | 459416.1 | 482991.5 | 421739.1 | 447232.1 | 537946.4 | 525148.5 | 0.2193959 | 2.117 |
| HPOD keto 36:4-PC-/18:2 | 1645985.4 | 1309743.6 | 1104565.2 | 1317500 | 1562410.7 | 302475.2 | 0.5212564 | 0.493 |
| HPOD keto 36:4-PC-/18:2 | 262408.8 | 321709.4 | 255108.7 | 266696.4 | 328303.6 | 294059.4 | 0.5798696 | 0.362 |
| HPOD keto 36:4-PE-/18:1 | 724671.5 | 889572.6 | 579565.2 | 953660.7 | 858660.7 | 1001782.2 | 0.1048355 | 4.368 |
| HPOD keto 36:4-PE-/18:2 | 232992.7 | 253589.7 | 254782.6 | 232321.4 | 244553.6 | 221287.1 | 0.2139485 | 2.179 |
| HPOD keto 36:4-PE-/18:3 | 138832.12 | 154786.32 | 113913.04 | 131696.43 | 153482.14 | 221683.17 | 0.3259398 | 1.251 |
| HPOD keto 36:4-PG-/18:1 | 565328.47 | 751623.93 | 824673.91 | 907589.29 | 804017.86 | 981089.11 | 0.1186833 | 3.924 |
| HPOD keto 36:4-PG-/18:2 | 153868.61 | 155128.21 | 189130.43 | 177500 | 191339.29 | 229801.98 | 0.1600161 | 2.968 |
| HPOD keto 36:5-PC-/18:2 | 252700.7 | 283247.9 | 224782.6 | 190267.9 | 290178.6 | 221980.2 | 0.597846 | 0.327 |
| HPOD keto 36:5-PC-/18:3 | 3274233.6 | 2904786.3 | 2831630.4 | 2606875 | 2870892.9 | 2522574.3 | 0.1227032 | 3.810 |
| HPOD keto 36:5-PC-/18:3 | 24598.5 | 28461.5 | 20000 | 23928.6 | 19732.1 | 16336.6 | 0.2558532 | 1.755 |
| HPOD keto 36:5-PE-/18:3 | 49927.007 | 57777.778 | 56956.522 | 80535.714 | 85892.857 | 85346.535 | 0.000651754 | 92.620 |
| HPOD keto 36:5-PE-/HPOT keto 36:4 PE | 254525.5 | 265384.6 | 203478.3 | 329553.6 | 233839.3 | 290891.1 | 0.265343 | 1.674 |
| HPOD keto 36:5-PG-/18:2 | 259562.04 | 325982.91 | 254021.74 | 280892.86 | 320714.29 | 356732.67 | 0.28169 | 1.546 |
| HPOD keto 36:6-PC-/18:3 | 947080.3 | 694871.8 | 864673.9 | 606339.3 | 794642.9 | 641683.2 | 0.1755711 | 2.702 |
| HPOT keto 34:2-PC-/16:0 | 660802.9 | 655982.9 | 600760.9 | 519375 | 651517.9 | 619901 | 0.3937338 | 0.912 |
| HPOT keto 34:2-PG-/16:0 | 526934.31 | 618632.48 | 576847.83 | 649107.14 | 735178.57 | 829801.98 | 0.04879957 | 7.841 |
| HPOT keto 34:3-PC-/16:0 | 53047518 | 49862906 | 44425326 | 47274554 | 47361250 | 47897426 | 0.5604839 | 0.402 |
| HPOT keto 34:3-PC-/16:0 | 117299.3 | 132307.7 | 78478.3 | 76071.4 | 110892.9 | 69703 | 0.3105298 | 1.346 |
| HPOT keto 34:3-PE-/16:0 | 25839.416 | 28547.009 | 47934.783 | 39017.857 | 66607.143 | 53960.396 | 0.1456039 | 3.253 |
| HPOT keto 34:3-PG-/16:0 | 151240.88 | 178034.19 | 220652.17 | 271875 | 266517.86 | 311980.2 | 0.01559205 | 16.330 |
| HPOT keto 36:4-PC-/18:1 | 193795.6 | 193418.8 | 154239.1 | 156428.6 | 185982.1 | 188316.8 | 0.8405656 | 0.046 |
| HPOT keto 36:4-PC-/18:2 | 3274233.6 | 2904786.3 | 1378804.3 | 1300000 | 1052053.6 | 1609405.9 | 0.1173185 | 3.964 |
| HPOT keto 36:4-PE-/18:1 | 56861.314 | 53076.923 | 55869.565 | 48214.286 | 45535.714 | 37425.743 | 0.02829819 | 11.290 |
| HPOT keto 36:4-PG-/18:1 | 261970.8 | 358888.89 | 328695.65 | 410625 | 380446.43 | 525445.54 | 0.08080388 | 5.400 |
| HPOT keto 36:4-PG-/18:2 | 144452.55 | 310940.17 | 125326.09 | 176517.86 | 284821.43 | 258712.87 | 0.5285003 | 0.475 |
| HPOT keto 36:5-PG-/18:2 | 35839.416 | 57521.368 | 42934.783 | 43928.571 | 56250 | 76336.634 | 0.3047591 | 1.383 |
| HPOT keto 36:6-PC-/18:3 | 34525.5 | 48974.4 | 30108.7 | 75089.3 | 26517.9 | 7722.8 | 0.94878 | 0.005 |
| LPC 13:0 | 49416.1 | 163675.2 | 314565.2 | 253839.3 | 229910.7 | 168118.8 | 0.6358375 | 0.262 |
| LPC 14:0 | 8076642.3 | 4993162.4 | 14069457 | 12130714 | 12435268 | 8329207.9 | 0.5539772 | 0.416 |
| LPC 15:0 | 746496.4 | 650170.9 | 1462826.1 | 807321.4 | 687678.6 | 702475.2 | 0.442365 | 0.725 |
| LPC 16:0 | 14026788 | 10416923 | 20962717 | 19103304 | 20641339 | 15737129 | 0.3812535 | 0.966 |
| LPC 16:1 | 39893796 | 28366752 | 63312174 | 60188661 | 51952946 | 39569307 | 0.6028051 | 0.318 |
| LPC 18:0 | 1140583.9 | 729145.3 | 1611195.7 | 1083660.7 | 1394464.3 | 645643.6 | 0.7401258 | 0.126 |
| LPC 18:1 | 39195839 | 26416581 | 60837391 | 58727232 | 50934464 | 37574257 | 0.588399 | 0.345 |
| LPC 18:2 | 14800365 | 10596752 | 18063478 | 15727679 | 15956607 | 12186238 | 0.9587349 | 0.003 |
| LPC 18:3 | 392700.7 | 422991.5 | 452391.3 | 496875 | 397946.4 | 394554.5 | 0.8599162 | 0.035 |
| LPC 20:0 | 15401.5 | 25470.1 | 14565.2 | 24375 | 199821.4 | 8415.8 | 0.3906945 | 0.925 |
| LPC 20:1 | 97153.3 | 23333.3 | 20326.1 | 75535.7 | 50178.6 | 68415.8 | 0.5352611 | 0.459 |
| LPC 20:2 | 16569.3 | 9230.8 | 16847.8 | 13035.7 | 72500 | 14653.5 | 0.3857165 | 0.946 |
| LPC 20:3 | 27956.2 | 28034.2 | 16521.7 | 17053.6 | 0 | 12178.2 | 0.08560258 | 5.159 |
| LPC 20:5 | 5839.4 | 72649.6 | 21739.1 | 43839.3 | 17767.9 | 0 | 0.617779 | 0.292 |
| LPC 22:1 | 20146 | 9658.1 | 34565.2 | 10892.9 | 16517.9 | 990.1 | 0.2324764 | 1.977 |
| LPC 22:6 | 9635 | 16410.3 | 19891.3 | 6160.7 | 17857.1 | 23267.3 | 0.9427165 | 0.006 |
| LPC 26:0 | 135328.5 | 96495.7 | 0 | 122767.9 | 92232.1 | 187920.8 | 0.3103567 | 1.347 |
| LPC(O-16:0) | 406204.4 | 106495.7 | 664021.7 | 753035.7 | 508839.3 | 401584.2 | 0.4451153 | 0.716 |
| LPC(O-18:0 | 344744.5 | 134871.8 | 777065.2 | 766160.7 | 504196.4 | 179207.9 | 0.8127114 | 0.064 |
| LPC(O-18:1) | 344890.5 | 322051.3 | 674565.2 | 651428.6 | 573750 | 434752.5 | 0.461139 | 0.663 |
| LPC(O-20:1) | 145839.4 | 77692.3 | 196304.3 | 165892.9 | 243303.6 | 94257.4 | 0.6394144 | 0.256 |
| LPC(O-24:2) | 94379.6 | 58205.1 | 67608.7 | 64553.6 | 27232.1 | 43762.4 | 0.1388797 | 3.402 |
| LPE(14:0)) | 1097737.2 | 777094 | 1574673.9 | 1436428.6 | 1166071.4 | 775445.5 | 0.9406096 | 0.006 |
| LPE(16:0) | 24217007 | 19993504 | 26377500 | 21817679 | 18649464 | 17911683 | 0.1413573 | 3.346 |
| LPE(18:0) | 3360438 | 2230769.2 | 3450869.6 | 1545357.1 | 2310000 | 2203663.4 | 0.09651274 | 4.681 |
| LPE(18:1) | 37572555 | 32011197 | 50273696 | 46774196 | 35402232 | 31949307 | 0.7989406 | 0.074 |
| LPE(18:2) | 5296277.4 | 4930341.9 | 6888587 | 5913125 | 4717589.3 | 4617524.8 | 0.4426274 | 0.725 |
| M34:2-PC-/16:0 | 395180876 | 363748034 | 351048261 | 342870268 | 342812589 | 336958218 | 0.09316898 | 4.818 |
| M34:2-PC-/16:0 | 394306.57 | 407094.02 | 522282.61 | 325982.14 | 387321.43 | 498514.85 | 0.5961013 | 0.331 |
| M34:2-PC-/18:2 | 1160292 | 1162991.5 | 1193260.9 | 1130803.6 | 1363839.3 | 1273465.3 | 0.2889831 | 1.492 |
| M34:2-PE-/16:0 | 428978.1 | 411452.99 | 599347.83 | 498035.71 | 554464.29 | 598217.82 | 0.3504465 | 1.116 |
| M34:2-PE-/18:2 | 431970.8 | 490854.7 | 535434.78 | 558839.29 | 787232.14 | 649108.91 | 0.06995686 | 6.034 |
| M34:2-PG-/16:0 | 87080.3 | 50598.3 | 86087 | 61428.6 | 64553.6 | 52970.3 | 0.2976693 | 1.431 |
| M34:2-PG-/18:2 | 84525.5 | 75897.4 | 59456.5 | 64732.1 | 102589.3 | 69405.9 | 0.7088742 | 0.161 |
| M34:3-PC-/16:0 | 205288248 | 179579316 | 164971304 | 144733661 | 158990714 | 144623366 | 0.05633544 | 7.081 |
| M34:3-PE-/16:0 | 31751.825 | 14102.564 | 43260.87 | 13482.143 | 28482.143 | 30198.02 | 0.6023434 | 0.319 |
| M36:4-PC-/18:1 | 39896058 | 35002821 | 33049022 | 23547321 | 28926875 | 26943960 | 0.02087534 | 13.670 |
| M36:4-PC-/18:2 | 332481.75 | 404358.97 | 355434.78 | 268928.57 | 421785.71 | 346930.69 | 0.7287801 | 0.138 |
| M36:4-PE-/18:2 | 489416.06 | 420000 | 553913.04 | 415535.71 | 515089.29 | 506930.69 | 0.8722579 | 0.029 |
| M36:4-PG-/18:2 | 39708 | 70683.8 | 51304.3 | 40357.1 | 51160.7 | 25445.5 | 0.2719967 | 1.620 |
| M36:5-PC-/18:3 | 2145036.5 | 1607948.7 | 1478695.7 | 977767.9 | 1344375 | 1143564.4 | 0.06260717 | 6.556 |
| M36:6-PC-/18:3 | 26277.4 | 21453 | 5543.5 | 29553.6 | 5625 | 20198 | 0.9439353 | 0.006 |
| modPC 540.5/0.78 | 92481.8 | 23418.8 | 154347.8 | 77678.6 | 115178.6 | 98415.8 | 0.8672662 | 0.032 |
| modPC 666.4/1.90 | 46058.4 | 71282.1 | 126413 | 48928.6 | 130625 | 42970.3 | 0.8573074 | 0.037 |
| modPC 843.6/7.10 | 3430.7 | 17265 | 20108.7 | 0 | 16607.1 | 39802 | 0.7017777 | 0.169 |
| oddPC 29:0 | 10380073 | 9096923.1 | 10458044 | 7985625 | 8461696.4 | 7681980.2 | 0.01754043 | 15.210 |
| oddPC 31:0 | 25436788 | 24147863 | 24158913 | 19602768 | 19123036 | 17848416 | 0.00106708 | 71.660 |
| oddPC 31:1 | 66959489 | 67651880 | 65388370 | 53069643 | 53174732 | 48502970 | 0.000850249 | 80.680 |
| oddPC 33:0 | 6205985.4 | 6853675.2 | 7004782.6 | 5535982.1 | 5872142.9 | 5309703 | 0.01929991 | 14.350 |
| oddPC 33:1 | 66628613 | 67228889 | 62993696 | 52742857 | 52192143 | 51158515 | 0.000635075 | 93.880 |
| oddPC 33:2 | 42559051 | 42484274 | 38779783 | 30696429 | 35250536 | 30897129 | 0.009766168 | 21.490 |
| oddPC 33:3 | 4135255.5 | 3081453 | 3306304.3 | 2100803.6 | 2673214.3 | 2546237.6 | 0.0428324 | 8.582 |
| oddPC 35:1 | 20165620 | 21617180 | 21501196 | 17508036 | 18260625 | 17546931 | 0.003213736 | 39.900 |
| oddPC 35:3 | 7914817.5 | 8185128.2 | 7362500 | 4722678.6 | 5345089.3 | 5648811.9 | 0.002097904 | 50.160 |
| oddPC 35:4 | 474233.6 | 471623.9 | 595543.5 | 295267.9 | 385625 | 328613.9 | 0.0218825 | 13.280 |
| oddPC 35:5 | 112481.8 | 20427.4 | 53369.6 | 96785.7 | 19642.9 | 50594.1 | 0.8635523 | 0.034 |
| oddPC 37:4 | 176350.4 | 205384.6 | 149239.1 | 124107.1 | 76517.9 | 93168.3 | 0.02088114 | 13.670 |
| oddPC 37:6 | 93795.6 | 115641 | 150000 | 114732.1 | 211071.4 | 81485.1 | 0.7244094 | 0.143 |
| oddPC 39:5 | 47591.2 | 30769.2 | 0 | 20357.1 | 8392.9 | 22673.3 | 0.5723056 | 0.377 |
| oddPC 39:6 | 148613.1 | 95726.5 | 29673.9 | 69196.4 | 22232.1 | 62574.3 | 0.345093 | 1.144 |
| oddPC 39:7 | 796496.4 | 665213.7 | 629347.8 | 590000 | 598750 | 735544.6 | 0.4673957 | 0.644 |
| oPDA 34:2-PG-/16:0 | 48613.139 | 69743.59 | 24239.13 | 45625 | 65178.571 | 57623.762 | 0.5802333 | 0.361 |
| oPDA 34:3-PC-/18:3 | 2557007.3 | 2462136.8 | 2601630.4 | 1867500 | 2110267.9 | 2009505 | 0.002615363 | 44.580 |
| PC 26:0 | 9043576.6 | 8403247.9 | 9340543.5 | 7820267.9 | 6904196.4 | 7282574.3 | 0.01421074 | 17.260 |
| PC 28:0 | 41012336 | 38012051 | 37600978 | 28233750 | 30338482 | 30657426 | 0.002270525 | 48.090 |
| PC 30:0 | 49890511 | 48509658 | 48320435 | 40671875 | 41163036 | 40201188 | 0.000131507 | 210.300 |
| PC 32:0 | 64124964 | 47951880 | 51744348 | 46508571 | 45863839 | 46619109 | 0.1657249 | 2.866 |
| PC 32:1 | 1.096E+09 | 949320598 | 894489891 | 823483750 | 800763839 | 772320495 | 0.04326788 | 8.523 |
| PC 32:2 | 714605766 | 759194103 | 686336957 | 619474018 | 587793929 | 578263564 | 0.007095336 | 25.780 |
| PC 32:3 | 7523941.6 | 6919572.6 | 6176195.7 | 4118482.1 | 5242857.1 | 4881386.1 | 0.01420326 | 17.260 |
| PC 34:0 | 39407080 | 29774872 | 29731848 | 26926607 | 26941161 | 23915446 | 0.1049374 | 4.364 |
| PC 34:1 | 526784234 | 455642650 | 418366196 | 373336696 | 369518839 | 372850891 | 0.04049888 | 8.916 |
| PC 34:2 | 722742263 | 662743419 | 590456739 | 536332054 | 546671161 | 524416139 | 0.03393236 | 10.030 |
| PC 34:3 | 111489927 | 97736410 | 81599783 | 57655982 | 66711607 | 63167723 | 0.01889716 | 14.540 |
| PC 34:4 | 16517445 | 12425128 | 12039022 | 8745357.1 | 10262679 | 8776138.6 | 0.04418801 | 8.401 |
| PC 34:5 | 22481.8 | 79572.6 | 27500 | 19285.7 | 13125 | 19207.9 | 0.2301068 | 2.001 |
| PC 36:0 | 4284744.5 | 3784273.5 | 3380760.9 | 3212767.9 | 2584464.3 | 3357128.7 | 0.09608195 | 4.698 |
| PC 36:1 | 39466496 | 36233504 | 31654891 | 30757411 | 32092946 | 30801782 | 0.1189434 | 3.916 |
| PC 36:2 | 208114891 | 182903077 | 166959674 | 149641339 | 139804018 | 149805644 | 0.03339886 | 10.140 |
| PC 36:3 | 60267372 | 49016838 | 44360544 | 35052946 | 37733839 | 35523663 | 0.03442903 | 9.939 |
| PC 36:4 | 111821752 | 81995385 | 71465109 | 46801161 | 56191964 | 57489505 | 0.0495952 | 7.753 |
| PC 36:5 | 109263723 | 87146325 | 78250870 | 48961964 | 57864732 | 56272673 | 0.01805269 | 14.950 |
| PC 36:6 | 168394.2 | 262393.2 | 85652.2 | 52410.7 | 58125 | 70594.1 | 0.09504217 | 4.740 |
| PC 38:2 | 1659708 | 1375641 | 980543.5 | 673392.9 | 1054732.1 | 1000990.1 | 0.1358585 | 3.472 |
| PC 38:3 | 193868.6 | 91025.6 | 146087 | 115178.6 | 247053.6 | 36930.7 | 0.8838343 | 0.024 |
| PC 38:4 | 18686.1 | 22478.6 | 45760.9 | 0 | 5714.3 | 32475.2 | 0.2829759 | 1.536 |
| PC 38:5 | 48175.2 | 31709.4 | 0 | 13125 | 15357.1 | 9405.9 | 0.3813321 | 0.966 |
| PC 38:6 | 160948.9 | 104615.4 | 73913 | 338482.1 | 248928.6 | 281485.1 | 0.008455633 | 23.340 |
| PC 38:7 | 287810.2 | 223418.8 | 296739.1 | 240982.1 | 212500 | 100297 | 0.1572595 | 3.020 |
| PC 40:5 | 40583.9 | 20341.9 | 12500 | 28303.6 | 22500 | 19108.9 | 0.9004058 | 0.018 |
| PC 40:6 | 45401.5 | 35299.1 | 30543.5 | 84464.3 | 47232.1 | 37821.8 | 0.2622913 | 1.700 |
| PC 40:7 | 98467.2 | 96068.4 | 136739.1 | 81964.3 | 27053.6 | 199207.9 | 0.8906324 | 0.021 |
| PC(O-32:2) | 7722043.8 | 8168803.4 | 6812500 | 4728750 | 5706339.3 | 4507920.8 | 0.008879353 | 22.700 |
| PC(O-34:4) | 485839.4 | 265384.6 | 352065.2 | 259553.6 | 84732.1 | 150396 | 0.06847504 | 6.133 |
| PC(O-36:0) | 3907080.3 | 2801880.3 | 2993478.3 | 2106071.4 | 2392321.4 | 2437326.7 | 0.06084011 | 6.695 |
| PC(O-36:2) | 18313796 | 17914872 | 14745870 | 9512410.7 | 12060000 | 11010891 | 0.01046839 | 20.640 |
| PC(P-30:0) | 4440365 | 4291880.3 | 4975543.5 | 4104642.9 | 3714017.9 | 3886435.6 | 0.04763074 | 7.975 |
| PC(P-36:5) | 735109.5 | 575128.2 | 514782.6 | 696517.9 | 525892.9 | 510297 | 0.7463084 | 0.120 |
| PE 32:0 | 17247956 | 17929487 | 16842717 | 14872946 | 14450714 | 15012178 | 0.00204525 | 50.840 |
| PE 32:1 | 237484818 | 243279658 | 244171196 | 206988839 | 209591071 | 197340495 | 0.000980205 | 74.920 |
| PE 34:0 | 43958759 | 41572137 | 46956413 | 35512679 | 35359375 | 37024257 | 0.007593431 | 24.810 |
| PE 34:1 | 401046861 | 389968291 | 434603696 | 354046607 | 346661339 | 348633960 | 0.0124396 | 18.670 |
| PE 34:2 | 346979051 | 334245214 | 380795217 | 297941250 | 290788571 | 292068119 | 0.01269138 | 18.450 |
| PE 34:3 | 76113577 | 64712650 | 72089130 | 52336429 | 58277857 | 49321188 | 0.01420512 | 17.260 |
| PE 35:1 | 12525256 | 12132992 | 15209565 | 9970803.6 | 11053125 | 11285941 | 0.07409138 | 5.776 |
| PE 35:2 | 25839416 | 27483932 | 32513044 | 23332500 | 22913036 | 23436634 | 0.05557629 | 7.150 |
| PE 36:0 | 5636861.3 | 4813076.9 | 5885652.2 | 4352321.4 | 4122589.3 | 4281089.1 | 0.02269401 | 12.980 |
| PE 36:1 | 57491971 | 52846325 | 60681196 | 51065000 | 52192857 | 52151188 | 0.08687463 | 5.098 |
| PE 36:2 | 157669051 | 159036154 | 181564891 | 145613214 | 142621071 | 153202970 | 0.08618244 | 5.131 |
| PE 36:3 | 70605110 | 68882992 | 78503370 | 54173661 | 58903482 | 54501584 | 0.007259783 | 25.450 |
| PE 36:4 | 20607299 | 18850513 | 20995326 | 12487946 | 13167054 | 14135050 | 0.001072643 | 71.470 |
| PE 36:5 | 2020438 | 1528205.1 | 1512608.7 | 825892.9 | 940803.6 | 911881.2 | 0.009557675 | 21.760 |
| PE 38:3 | 563649.6 | 759829.1 | 684347.8 | 500803.6 | 482767.9 | 472079.2 | 0.03329404 | 10.160 |
| PE 38:4 | 24598.5 | 13846.2 | 23587 | 0 | 29732.1 | 16336.6 | 0.5961335 | 0.331 |
| PE 40:7 | 37153.3 | 43846.2 | 14130.4 | 0 | 0 | 10099 | 0.04197704 | 8.701 |
| PE(O-18:1/18:2) | 9493868.6 | 7559401.7 | 11073152 | 8231339.3 | 7616785.7 | 7541089.1 | 0.2032972 | 2.308 |
| PE(O-18:2/18:2) | 41897.8 | 153418.8 | 193369.6 | 46517.9 | 52053.6 | 43168.3 | 0.1440109 | 3.288 |
| PE(O-34:1) | 8661386.9 | 8199914.5 | 10076630 | 7716964.3 | 8143839.3 | 6629306.9 | 0.1094467 | 4.211 |
| PE(O-34:2) | 6361678.8 | 5982649.6 | 7311739.1 | 4888125 | 5676071.4 | 4957425.7 | 0.04238376 | 8.644 |
| PE(O-36:2) | 27859562 | 29841624 | 34486087 | 23993929 | 24490000 | 24988317 | 0.03473333 | 9.881 |
| PE(O-36:5) | 19854 | 19914.5 | 41304.3 | 22053.6 | 13571.4 | 7425.7 | 0.2016655 | 2.329 |
| PE(O-36:6) | 832919.7 | 690427.4 | 813587 | 472053.6 | 636696.4 | 452277.2 | 0.02452114 | 12.370 |
| PE(P-34:1) | 6361678.8 | 5982649.6 | 7311739.1 | 4888125 | 5676071.4 | 4957425.7 | 0.04238376 | 8.644 |
| PE(P-34:2) | 76569.3 | 58717.9 | 24782.6 | 49017.9 | 9821.4 | 37722.8 | 0.3307738 | 1.223 |
| PE(P-36:1) | 25839416 | 27483932 | 32513044 | 23384196 | 22913036 | 23436634 | 0.05611401 | 7.101 |
| PE(P-36:2) | 9493868.6 | 7559401.7 | 11271957 | 8231339.3 | 7616785.7 | 7541089.1 | 0.2070589 | 2.262 |
| PE(P-38:5) | 140219 | 142820.5 | 68804.3 | 39107.1 | 81071.4 | 32178.2 | 0.08112035 | 5.383 |
| PE(P-38:6) | 4187372.3 | 3996495.7 | 4022608.7 | 3143839.3 | 3957767.9 | 3114455.4 | 0.07879739 | 5.507 |
| PE(P-40:6) | 2060802.9 | 2177777.8 | 1853587 | 1458750 | 1420892.9 | 1367227.7 | 0.00334377 | 39.050 |
| PG 34:0 | 1762481.8 | 1901623.9 | 1815217.4 | 1444107.1 | 1196517.9 | 1467227.7 | 0.00878318 | 22.840 |
| PG 34:1 | 12057226 | 10262821 | 9922717.4 | 8390625 | 7864732.1 | 8854851.5 | 0.03003732 | 10.870 |
| PG 36:1 | 1354744.5 | 1532307.7 | 1240108.7 | 1013303.6 | 889285.7 | 860198 | 0.009422124 | 21.940 |
| PG 36:2 | 7116569.3 | 6103418.8 | 5485652.2 | 5169732.1 | 3989821.4 | 4499009.9 | 0.04530112 | 8.258 |
| PI 32:0 | 3993284.7 | 2875555.6 | 2184021.7 | 2248303.6 | 2382232.1 | 2093465.3 | 0.2194537 | 2.116 |
| PI 32:1 | 40995183 | 36499573 | 28978370 | 28570179 | 24804107 | 25120990 | 0.06565587 | 6.329 |
| PI 34:1 | 36341168 | 31234615 | 26673152 | 24915446 | 22990179 | 22066139 | 0.05005534 | 7.703 |
| PI 36:2 | 33692117 | 27380427 | 23406087 | 21551518 | 21750982 | 20047723 | 0.08160964 | 5.358 |
| PI 36:3 | 56620584 | 44548974 | 35233044 | 28327679 | 30646250 | 28564059 | 0.05926568 | 6.825 |
| PI 36:4 | 9890365 | 8441709.4 | 6787282.6 | 5392321.4 | 6050625 | 4503861.4 | 0.03800073 | 9.307 |
| PI 38:2 | 784890.5 | 634017.1 | 568152.2 | 567589.3 | 502232.1 | 575445.5 | 0.1702278 | 2.789 |
| PI 38:3 | 363065.7 | 291111.1 | 267065.2 | 236339.3 | 274732.1 | 192475.2 | 0.1241131 | 3.771 |
| PI 38:4 | 20656.9 | 23931.6 | 3260.9 | 35357.1 | 55982.1 | 47623.8 | 0.02580159 | 11.980 |
| PI 38:5 | 13868.6 | 6923.1 | 1087 | 982.1 | 9375 | 990.1 | 0.4908351 | 0.574 |
| PS 34:0 | 1448175.2 | 1458034.2 | 536413 | 427142.9 | 504910.7 | 264257.4 | 0.07541851 | 5.697 |
| PS 36:1 | 19808832 | 15226667 | 4062282.6 | 4888660.7 | 5184910.7 | 4778514.9 | 0.1591018 | 2.985 |
| PS 36:2 | 47652920 | 38141197 | 11469783 | 13370714 | 15620000 | 12785050 | 0.1638741 | 2.899 |
| PS 38:3 | 353211.7 | 206581.2 | 0 | 62946.4 | 136339.3 | 52772.3 | 0.3870919 | 0.940 |
| PS 38:4 | 33138.7 | 51111.1 | 26195.7 | 446.4 | 625 | 26237.6 | 0.07086002 | 5.976 |
| TG 14:0 16:0 18:2 | 630494526 | 669751966 | 759477391 | 581729911 | 568705804 | 571942970 | 0.04279537 | 8.587 |
| TG 14:0 16:1 18:1 | 535521898 | 549114444 | 615328696 | 479630625 | 465754107 | 510458119 | 0.04369724 | 8.466 |
| TG 14:0 16:1 18:2 | 74904015 | 76900940 | 93190109 | 61805625 | 70173304 | 66145347 | 0.0675003 | 6.199 |
| TG 14:0 18:0 18:1 | 59737883 | 66918974 | 35529348 | 58514554 | 22126339 | 22946436 | 0.270761 | 1.630 |
| TG 14:0 18:2 18:2 | 230948.9 | 152906 | 236087 | 96607.1 | 241607.1 | 226930.7 | 0.7493702 | 0.117 |
| TG 14:1 16:0 18:1 | 40544380 | 46924957 | 133421848 | 104736071 | 91062500 | 104773168 | 0.4301815 | 0.768 |
| TG 14:1 16:1 18:0 | 1.567E+09 | 1.696E+09 | 1.83E+09 | 1.446E+09 | 1.34E+09 | 1.432E+09 | 0.02454911 | 12.360 |
| TG 14:1 18:0 18:2 | 2523065.7 | 2262991.5 | 3556521.7 | 3186696.4 | 6131607.1 | 3363564.4 | 0.2337074 | 1.964 |
| TG 14:1 18:1 18:1 | 32043.8 | 427350.4 | 108804.3 | 159285.7 | 18660.7 | 188019.8 | 0.6360887 | 0.261 |
| TG 15:0 18:1 16:0 | 108613.1 | 0 | 8587 | 71875 | 51160.7 | 12475.2 | 0.8831162 | 0.025 |
| TG 15:0 18:1 18:1 | 675401.5 | 627777.8 | 616087 | 445535.7 | 316785.7 | 491881.2 | 0.01613838 | 15.990 |
| TG 16:0 16:0 16:0 | 97078540 | 109914957 | 114342283 | 103131071 | 88310625 | 80965248 | 0.1216602 | 3.839 |
| TG 16:0 16:0 18:0 | 21970365 | 27405043 | 26078370 | 23567143 | 11130357 | 10018218 | 0.09184627 | 4.875 |
| TG 16:0 16:0 18:1 | 3375036.5 | 4664957.3 | 4567282.6 | 2775892.9 | 3246339.3 | 3252277.2 | 0.06648643 | 6.270 |
| TG 16:0 16:0 18:2 | 6980365 | 6661111.1 | 20597391 | 6279196.4 | 14378661 | 13268812 | 0.9851243 | 0.000 |
| TG 16:0 16:1 18:1 | 5438394.2 | 6583418.8 | 6842608.7 | 5629375 | 5803392.9 | 6096039.6 | 0.3807717 | 0.968 |
| TG 16:0 18:0 18:1 | 59024380 | 108557094 | 58420544 | 53022768 | 34311964 | 36052079 | 0.1246996 | 3.755 |
| TG 16:0 18:1 18:1 | 4514671.5 | 5233162.4 | 5572173.9 | 4505714.3 | 4380446.4 | 6134059.4 | 0.8844064 | 0.024 |
| TG 16:0 18:1 18:2 | 327445.3 | 400085.5 | 417717.4 | 302767.9 | 359464.3 | 305742.6 | 0.1497534 | 3.167 |
| TG 16:0 18:2 18:2 | 164160.6 | 251623.9 | 122717.4 | 222232.1 | 170178.6 | 231386.1 | 0.5402058 | 0.447 |
| TG 16:1 16:1 16:1 | 544308905 | 561594786 | 630601957 | 504186339 | 493976607 | 519528119 | 0.05619388 | 7.093 |
| TG 16:1 16:1 18:0 | 28997226 | 31229145 | 42371739 | 28500625 | 29610982 | 35104158 | 0.5349839 | 0.460 |
| TG 16:1 16:1 18:1 | 32561314 | 36603248 | 43274783 | 33790625 | 34746518 | 39146733 | 0.6768423 | 0.201 |
| TG 16:1 18:1 18:1 | 35266423 | 35700855 | 49396413 | 36790893 | 38025000 | 47227525 | 0.9263327 | 0.010 |
| TG 16:1 18:1 18:2 | 2711824.8 | 2434444.4 | 5661195.7 | 2660892.9 | 11846518 | 5588712.9 | 0.345706 | 1.141 |
| TG 17:0 16:0 16:1 | 76938029 | 98935385 | 84180326 | 76551696 | 72030089 | 75574455 | 0.144803 | 3.271 |
| TG 17:0 16:0 18:0 | 3445620.4 | 6447008.5 | 3920000 | 4506964.3 | 4204910.7 | 4150792.1 | 0.7526708 | 0.114 |
| TG 17:0 17:0 17:0 | 3174671.5 | 4165470.1 | 2765326.1 | 3332321.4 | 3427321.4 | 3391584.2 | 0.9725453 | 0.001 |
| TG 17:0 18:1 14:0 | 19571971 | 24934188 | 21418044 | 19981339 | 18069464 | 19664753 | 0.1787818 | 2.652 |
| TG 17:0 18:1 16:0 | 12858248 | 18501709 | 15449239 | 12354107 | 19445179 | 14553069 | 0.9570205 | 0.003 |
| TG 17:0 18:1 16:1 | 15497153 | 16956496 | 17988478 | 14367946 | 14165089 | 17637822 | 0.3470592 | 1.133 |
| TG 17:0 18:1 18:1 | 855182.5 | 997435.9 | 820760.9 | 1189196.4 | 778214.3 | 940000 | 0.5841013 | 0.354 |
| TG 17:0 18:2 16:0 | 27540073 | 32246325 | 28521630 | 24370089 | 24639643 | 26549505 | 0.05556407 | 7.151 |
| TG 18:0 18:0 18:0 | 304525.5 | 271196.6 | 322173.9 | 317767.9 | 170446.4 | 338118.8 | 0.6863361 | 0.189 |
| TG 18:0 18:0 18:1 | 1717080.3 | 3172222.2 | 1811195.7 | 3300892.9 | 2347857.1 | 1529108.9 | 0.8301572 | 0.052 |
| TG 18:0 18:1 18:1 | 3664671.5 | 11226239 | 3179130.4 | 2476517.9 | 2002767.9 | 2120495 | 0.2166787 | 2.147 |
| TG 18:0 18:2 18:2 | 40219 | 11282.1 | 34565.2 | 34642.9 | 18214.3 | 130792.1 | 0.4197964 | 0.807 |
| TG 18:1 14:0 16:0 | 485358029 | 561037949 | 554505326 | 498236250 | 413928393 | 414283960 | 0.06900065 | 6.097 |
| TG 18:1 18:1 18:1 | 250656.9 | 229658.1 | 290760.9 | 91250 | 199285.7 | 132079.2 | 0.03273679 | 10.270 |
| TG 18:1 18:1 18:2 | 207518.2 | 96666.7 | 62500 | 49821.4 | 19285.7 | 224752.5 | 0.7698867 | 0.098 |
| TG 18:1 18:2 18:2 | 2992.7 | 37179.5 | 10000 | 0 | 0 | 0 | 0.1839589 | 2.573 |
| TG 48:0 | 46913942 | 52399915 | 47269674 | 39552500 | 33949732 | 33776139 | 0.007249388 | 25.470 |
| TG 48:1 | 164514015 | 177732821 | 186932174 | 148161786 | 132334732 | 132367228 | 0.009802697 | 21.440 |
| TG 48:2 | 80404599 | 76567350 | 85193261 | 63469911 | 61652500 | 67153366 | 0.005018251 | 31.270 |
| TG 48:3 | 36990073 | 40431966 | 41958370 | 31656429 | 32390625 | 35573465 | 0.02559527 | 12.040 |
| TG 49:1 | 6306934.3 | 5606068.4 | 6853804.3 | 5206964.3 | 5459910.7 | 6153168.3 | 0.2300537 | 2.002 |
| TG 50:0 | 27762847 | 33635983 | 29193478 | 24392679 | 9178571.4 | 10506931 | 0.04013028 | 8.971 |
| TG 50:1 | 106276204 | 115199915 | 61127609 | 93400000 | 40514286 | 43302079 | 0.2169026 | 2.145 |
| TG 50:2 | 64464380 | 68555470 | 72122500 | 58767411 | 52781696 | 65158218 | 0.08714236 | 5.086 |
| TG 50:3 | 20103723 | 20226239 | 23291739 | 18187768 | 17085268 | 20766535 | 0.1692936 | 2.805 |
| TG 50:4 | 556934.3 | 1131880.3 | 1488804.3 | 910714.3 | 3175178.6 | 1605643.6 | 0.3107319 | 1.345 |
| TG 51:0 | 952116.8 | 1751709.4 | 1355434.8 | 1456160.7 | 1380982.1 | 1535247.5 | 0.6800052 | 0.197 |
| TG 51:2 | 4082408.8 | 3913247.9 | 4600434.8 | 2924642.9 | 3567500 | 4434455.4 | 0.3141488 | 1.323 |
| TG 52:1 | 17029562 | 27634274 | 16381630 | 15477232 | 10756518 | 14801980 | 0.1652533 | 2.874 |
| TG 52:2 | 18027226 | 19326068 | 22579565 | 15288304 | 7028482.1 | 19112772 | 0.1811814 | 2.615 |
| TG 52:4 | 233941.6 | 283418.8 | 284565.2 | 222410.7 | 827232.1 | 382376.2 | 0.3120675 | 1.336 |
| TG 53:2 | 16276350 | 28321111 | 18355544 | 14912321 | 15642679 | 17134555 | 0.2489284 | 1.817 |
| TG 54:1 | 594087.6 | 828974.4 | 395652.2 | 621785.7 | 68928.6 | 100891.1 | 0.1924001 | 2.452 |
| TG 54:2 | 9854 | 35299.1 | 7500 | 37857.1 | 14821.4 | 19207.9 | 0.6026968 | 0.318 |
| TG 54:3 | 1263211.7 | 1341025.6 | 1254456.5 | 1124285.7 | 1370535.7 | 1770198 | 0.5157417 | 0.507 |
| TG 54:4 | 276204.4 | 135213.7 | 39565.2 | 274107.1 | 339821.4 | 200099 | 0.2035415 | 2.305 |
| TG 54:5 | 172627.7 | 133589.7 | 86630.4 | 51160.7 | 93482.1 | 46039.6 | 0.0811771 | 5.380 |
| TG 54:6 | 199124.1 | 232051.3 | 338587 | 206250 | 203660.7 | 211782.2 | 0.3066598 | 1.371 |
| TG 56:6 | 76715.3 | 142649.6 | 244347.8 | 86071.4 | 9107.1 | 136930.7 | 0.2763682 | 1.586 |
| TG 56:8 | 518978.1 | 423589.7 | 645108.7 | 423482.1 | 379107.1 | 515940.6 | 0.3019053 | 1.402 |
